# Ultrasound composite scores for the assessment of inflammatory and structural pathologies in Psoriatic Arthritis (PsASon-Score)

**DOI:** 10.1186/s13075-014-0476-2

**Published:** 2014-10-31

**Authors:** Anja Ficjan, Rusmir Husic, Judith Gretler, Angelika Lackner, Winfried B Graninger, Marwin Gutierrez, Christina Duftner, Josef Hermann, Christian Dejaco

**Affiliations:** Department of Rheumatology and Immunology, Medical University Graz, Auenbruggerplatz 15, 8036 Graz, Austria; Clinica Reumatologica, Università Politecnica delle Marche, Ospedale “Carlo Urbani”, Via dei Colli 52, 60035 Jesi Ancona, Italy; Department of Internal Medicine VI, Medical University Innsbruck, Anichstrasse 35, 6020 Innsbruck, Austria

## Abstract

**Introduction:**

This study was performed to develop ultrasound composite scores for the assessment of inflammatory and structural lesions in Psoriatic Arthritis (PsA).

**Methods:**

We performed a prospective study on 83 PsA patients undergoing two study visits scheduled 6 months apart. B-mode and Power Doppler (PD) findings were semi-quantitatively scored at 68 joints (evaluating synovia, perisynovial tissue, tendons and bone) and 14 entheses. We constructed bilateral and unilateral (focusing the dominant site) ultrasound composite scores selecting relevant sites by a hierarchical approach. We tested convergent construct validity, reliability and feasibility of inflammatory and structural elements of the scores as well as sensitivity to change for inflammatory items.

**Results:**

The bilateral score (termed PsASon22) included 22 joints (6 metacarpophalangeal joints (MCPs), 4 proximal interphalangeal joints (PIPs) of hands (H-PIPs), 2 metatarsophalangeal joints (MTPs), 4 distal interphalangeal joints (DIPs) of hands (H-DIPs), 2 DIPs of feet (F-DIPs), 4 large joints) and 4 entheses (bilateral assessment of lateral epicondyle and distal patellar tendon). The unilateral score (PsASon13) compromised 13 joints (2 MCPs, 3 H-PIPs, 1 PIP of feet (F-PIP), 2 MTPs, 1 H-DIP and 2 F-DIPs and 2 large joints) and 2 entheses (unilateral lateral epicondyle and distal patellar tendon). Both composite scores revealed a moderate to high sensitivity (bilateral composite score 43% to 100%, unilateral 36% to 100%) to detect inflammatory and structural lesions compared to the 68-joint/14-entheses score. The inflammatory and structural components of the composite scores correlated weakly with clinical markers of disease activity (corr_coeffs_ 0 to 0.40) and the health assessment questionnaire (HAQ, corr_coeffs_ 0 to 0.39), respectively. Patients with active disease achieving remission at follow-up yielded greater reductions of ultrasound inflammatory scores than those with stable clinical activity (Cohen’s d effect size ranging from 0 to 0.79). Inter-rater reliability of bi- and unilateral composite scores was moderate to good with ICCs ranging from 0.42 to 0.96 and from 0.36 to 0.71, respectively for inflammatory and structural sub-scores. The PsASon22 and PsASon13 required 16 to 26 and 9 to 13 minutes, respectively to be completed.

**Conclusion:**

Both new PsA ultrasound composite scores (PsASon22 and PsASon13) revealed sufficient convergent construct validity, sensitivity to change, reliability and feasibility.

**Electronic supplementary material:**

The online version of this article (doi:10.1186/s13075-014-0476-2) contains supplementary material, which is available to authorized users.

## Introduction

Current EULAR recommendations on the use of imaging techniques in rheumatoid arthritis (RA) recognize the high sensitivity of sonography to detect joint pathologies, suggesting the use of this technique for a more accurate assessment of patients’ disease activity as compared to clinical examination alone [1]. In routine practice and clinical trials of psoriatic arthritis (PsA), disease activity is still monitored by RA-specific clinical composite scores [2]. These measures, however, are of questionable value for PsA because of the heterogeneous nature of the disease characterized by various articular and extra-articular manifestations [3].

We recently reported good performance of sonography for the assessment of disease activity in PsA as determined by the investigation of 68 synovial sites and periarticular structures, as well as 14 entheses [4]. A comprehensive ultrasound assessment as performed in this study, however, is not feasible in daily routine practice, whereas a reduced ultrasound composite score might enable sonographic scoring of PsA patients in interventional trials and clinical practice.

A single ultrasound composite score has been developed for the assessment of PsA patients so far: the Italian so-called five-targets score focuses on joints, tendons, entheses, skin and nails; however, as only one site is assessed for each item, this index is of limited value to determine overall disease activity [5]. The German U7 score, primarily developed for RA, has occasionally been used to monitor disease activity in PsA patients in interventional studies; however, this score does not include important PsA manifestations such as enthesitis or distal interphalangeal joint (DIP) arthritis [6,7].

The aim of this study was to develop ultrasound composite scores that (1) include all currently defined ultrasound pathologies of PsA, (2) are sensitive for detection of inflammation and structural damage as compared to the assessment of 68 joints and 14 entheses and (3) are feasible in clinical practice. We tested the construct validity, reliability and feasibility of inflammatory and structural elements of the new composite scores and investigated the sensitivity to change for the inflammatory items.

## Materials and methods

### Patients

We performed a prospective study on 83 consecutive PsA patients between July 2011 and May 2013. All patients fulfilled the classification for psoriatic arthritis (CASPAR) criteria and had peripheral articular manifestations [8]. The institutional review board of the Medical University Graz approved the study and written informed consent was obtained from each patient.

Two study visits were scheduled 6 months apart. At each visit, complete history and clinical assessments were performed by one of three rheumatologists (AF, JG and JH) unaware of the ultrasound results [4]. The following parameters were recorded: number of tender joints (TJ) and swollen joints (SJ) according to the 66/68 articular index, presence of enthesitis according to the Leeds enthesitis index (LEI) and a clinical counterpart of the Madrid sonographic enthesis index (MASEI) plus lateral epicondyle (cMASEI + E) [9,10]. Patients’ global assessment of disease activity (PGA), patients’ pain assessment (Ptpain) and the evaluator’s global assessment of disease activity (EGA) were determined on visual analogue scales (range 0 to 100 mm). We also recorded patients’ questionnaires as previously described [4]. Blood samples were routinely tested for erythrocyte sedimentation rate (ESR, range 0 to 10 mm/first hour) and C-reactive protein (CRP) (range 0 to 5 mg/L).

We calculated the following clinical composite scores: the disease activity index for psoriatic arthritis (DAPSA), the composite psoriatic disease activity index (CPDAI) and a modified psoriatic arthritis disease activity score (PASDAS) omitting the short form health survey 36 (SF-36) component, which was not available in our patients [11-13]. In addition, we applied the minimal disease activity (MDA) criteria (5 of the 7 following items: TJ ≤1, SJ ≤1, Psoriasis activity and severity index (PASI) ≤1, Ptpain ≤15 mm, PGA ≤20 mm, health assessment questionnaire (HAQ) ≤0.5, tender enthesal points ≤1]) [14] and asked the evaluating rheumatologist for an overall judgment of patients’ clinical disease activity without using formal criteria or scores (two possible levels: active disease or remission).

### Ultrasound protocol

Sonographic evaluations were performed by one of two rheumatologists (CDe, RH) at the same day of clinical investigation as previously described in detail [4]. Briefly, grey scale (GS) and power Doppler (PD) sonography were performed at 68 joints and 14 entheses using a MyLab Twice ultrasound device (Esaote, Genova, Italy) with two multifrequence linear transducers (6 to 18 MHz and 4 to 13 MHz). GS synovitis (GSS) and PD-signals at joints (PD-j) were subjectively graded from 0 to 3 [15-17]. Perisynovitis was investigated by dorsal scans of metacarpophalangeal joints (MCP) 2 to 5 and was graded in GS (GS-perisyn) and PD (PD-perisyn) with 0 = absent or 1 = present [18]. Tenosynovitis was identified in GS (GS-teno) and graded from 0 to 3 (wrists, ankles) or with 0 = absent or 1 = present at small joints. PD-signals related to tenosynovitis (PD-teno) were graded from 0 to 3. Erosions or osteophytes were also semiquantitatively graded from 0-3 [4].

Enthesitis was assessed according to the MASEI investigating the presence and/or extent of erosions, enthesophytes, PD-signals (PD-e) and GS-changes [9]. We considered GS-changes of enthesitis (GSE) as a combined feature including the loss of fibrillar pattern, hypoechoic aspect, bursitis (infrapatellar and/or retrocalcaneal bursa) and/or enthesal thickening. The following anatomical sites were scanned: the insertion of the common extensor tendon at the lateral epicondyle, the distal insertion of the triceps into the olecranon, the quadriceps insertion into the upper pole of the patella, the patellar tendon insertion into the lower pole of the patella and into the tibial anterior tuberosity, the insertion of the Achilles tendon as well as the insertion of plantar aponeurosis into the calcaneal bone [4]. GSS/GSE refers to GSS and GSE and PD-j/e combines PD-j and PD-e scores.

### Construction of the ultrasound composite scores

Our aim was to develop ultrasound composite scores (1) that corroborate all currently defined ultrasound pathologies in PsA including GSS, GSE, PD-j, PD-e, GS-perisyn, PD-perisyn, GS-teno, PD-teno, erosions and ostheophytes/enthesophytes at small, middle/large and DIP joints as well as entheses (as appropriate); (2) that have a high sensitivity to detect these PsA characteristic ultrasound features as compared to the comprehensive assessment of 68 joints and 14 entheses; and (3) whose completion is feasible in clinical practice.

We used the 68-joints/14-entheses ultrasound score as a reference for the development of the new composite scores acknowledging that this instrument has not been validated for monitoring PsA patients yet. We constructed a bilateral and a unilateral composite score (focusing on the dominant site) using the Spanish 12-joint and the German 7-joint scores, respectively, as examples [6,19]. For the inclusion of relevant joints and entheses, we applied a hierarchical approach as exemplarily depicted in Additional file 1 and described in the following paragraph.

We conducted separate cycles of the procedure for each of the following anatomical regions and ultrasound pathologies: (1) small joints (defined as MCPs, metatarsophalangeal joints (MTP) as well as proximal interphalangeal joints (PIP) of hands (H-PIP) and feet (F-PIP)): GSS, PD-j, GS-perisyn (MCPs only), PD-perisyn (MCPs only), GS-teno, PD-teno, erosions and osteophytes; (2) DIP joints of the hand (H-DIP) and feet (F-DIP): GSS, PD-j, GS-teno, PD-teno, erosions and osteophytes; (3) large joints (defined as wrists, elbows, shoulders, hips, knees and ankles): GSS, PD-j, GS-teno (wrists and ankles only) and PD-teno (wrists and ankles only); and (4) entheses: GSE, PD-e, erosions and enthesophytes.

Each cycle started with the identification of the joint/enthesis most commonly revealing the ultrasound lesion of interest (for example, the joint that most frequently showed PD-positivity among small joints - MTP1). For the subsequent procedure, we excluded patients with a positive result at this step (in the example: patients with PD signals at MTP1). Among remaining cases, we identified the most frequently affected joint/enthesis again (in the example: cases with a positive PD result at MCP2). The rationale for excluding patients (and not simply searching for the second most commonly affected site among all cases) was to eliminate strong correlations between joint/enthesis, thus, maximizing the gain of sensitivity by any new site. We repeated this procedure n-times until a combination of sites reached ≥90% sensitivity to detect the corresponding ultrasound abnormality as compared to the 68-joint/14-entheses score. In case the gain of sensitivity by the inclusion of a new item was <20% or <10% for the bilateral or unilateral composite scores, respectively, or the overall prevalence of the finding at a given site was <5%, the selection process was terminated earlier. The rationale for premature termination was the prevention of mechanistic inclusion of sites with a negligible contribution to the overall performance of the composite scores potentially limiting the feasibility of the scores.

Next, we tested whether dorsal or palmar/plantar scans at H-PIPs, H-DIPs, MTPs, F-PIPs, F-DIPs and wrists, as well as medial/lateral or suprapatellar scans at knees, were dispensable for the composite scores. For this purpose, we analysed the proportion of ultrasound abnormalities exclusively detected by dorsal or palmar/plantar scans as well as by medial/lateral or suprapatellar scans of knees. Scans with a yield of <20% were omitted. MCPs were not subject to this analysis, because both palmar and dorsal scans are required to investigate the composite core elements peri- and tenosynovitis, respectively.

### Statistical analysis and validation of the ultrasound composite scores

Statistical analysis was performed using SPSS (version 20.0). Descriptive statistics were used to summarize the data. For continuous non-parametric data, we show the median and range whereas for parametric data, the mean and standard deviation are depicted. Comparisons between independent groups were conducted using the Mann-Whitney *U*-test and paired data were analysed with the Wilcoxon test (non-parametric data) or Student’s *t*-test (parametric data). Paired categorical data were analysed with the McNemar test.

For validation, we tested inflammatory (that is, GSS/GSE, PD-j/e, GS-Peri, PD-Peri, GS-Teno, PD-Teno) and structural elements (erosions, osteophytes/enthesophytes) of the new ultrasound scores separately. In addition, we constructed a global ultrasound inflammation subscore (GUIS) adding the results of GSS/GSE, PD-j/e, GS-perisyn, PD-perisyn, B-teno and PD-teno.

Convergent construct validity was investigated by the correlation (using Spearman’s rank correlation test) of inflammatory items (including GUIS) with clinical parameters of disease activity, and by correlating structural components with the HAQ (analysing the total cohort as well as patients in clinical remission separately in order to adjust for the activity related component of disability [20]).

Sensitivity to change was determined for the inflammatory components (including GUIS) only, as for structural elements the 6-months follow-up period was considered to be too short for the detection of any alterations. We compared the changes of the inflammatory subscore from baseline to the 6-months follow-up visit between patients with stable clinical disease activity (that is, active or inactive disease at both visits) and those being active at baseline and achieving remission (as determined by the evaluating physician) or MDA at follow up. We also calculated Cohen’s *d* effect-size statistic and the standardized response means (SRMs) of the total cohort versus patients in whom clinical disease activity improved [21]. Changes of the inflammatory elements were additionally correlated with alterations of clinical composite scores and its components. Inter-rater reliability of the ultrasound composite scores was determined by serial assessments of 10% of patients by two investigators (CDe, RH) and using the intraclass correlation coefficient (ICC).

## Results

### Clinical findings at baseline and follow-up visits

Patients’ characteristics are summarized in Table 1. At baseline, 40 patients (48.2%) were in remission as judged by the evaluating physician and 28 patients (33.7%) fulfilled the MDA criteria. Of the 83 patients 13 (15.7%) did not complete 6-month follow up. At follow up, 41 patients (58.6%) had stable disease activity according to physician’s evaluation (25 (35.7%) were inactive and 16 (22.9%) were active at both time points), 21 (30.0%) changed from active disease to remission and 3 (4.3%) from remission to active disease. Applying the MDA criteria, 15 of those (27.3%) being active at baseline reached MDA at follow up, 7 (25.0%) with MDA at baseline did not fulfill these criteria at the second visit, and 48 patients (68.6%) had stable disease (that is, were active (n =32) or had MDA (n =16) at both visits).

**Table 1:** **Clinical findings at baseline and 6-month follow up in patients with psoriatic arthritis**

	**Baseline visit (n =83)**	**Follow up at 6 months (n =70)**	***P*** **-value**
Age at inclusion, years^†^	51.8 (11.7)	n.a.	n.a.
Female, n (%)	22 (26.2)	n.a.	n.a.
Body mass index, kg/m^2‡^	27.2 (18.5 to 46.8)	n.a.	n.a.
Disease duration, years^‡^	7.5 (0 to 44.7)	n.a.	n.a.
Patients with a new diagnosis^¥^, n (%)	4 (4.8)	n.a.	n.a.
Axial involvement, n (%)	11 (13.3)	n.a.	n.a.
Smokers, n (%)		n.a.	n.a.
Current	21 (25.3)		
Previous	32 (38.6)		
PASDAS‡	5.9 (1.5)	5.2 (1.5)	<0.001
DAPSA‡	11.5 (0.1 to 70.2)	7.2 (0.1 to 73.5)	0.016
CPDAI‡	3.0 (0 to 9.0)	2.0 (0 to 11.0)	0.019
CRP, mg/L ‡	2.2 (0 to 49.5)	2.4 (0.2 to 35.0)	n.s.
ESR, mm/1st hour‡	9.5 (1 to 74)	10 (2 to 51)	n.s.
TJ, 68-joint count‡	4 (0 to 59)	1 (0 to 53)	0.012
SJ, 66-joint count‡	1 (0 to 15)	0 (0 to 17)	0.011
PGA, mm†	31.2 (22.7)	21.3 (23.5)	0.073
Ptpain, mm†	30.5 (24.5)	20.7 (21.0)	<0.001
EGA, mm†	22.0 (19.1)	11.5 (14.6)	0.009
HAQ†	0.7 (0.8)	0.6 (0.7)	<0.001
BASDAI‡	4.7 (0.9 to 5.5)	3.0 (0 to 6.0)	n.s.
ASQol‡	2.0 (0 to 11.0)	4.0 (0 to 8.0)	n.s.
Leeds enthesitis score‡	0 (0 to 4)	0 (0 to 2)	n.s.
Dactylitis score‡	0 (0 to 10)	0 (0 to 4)	n.s.
PASI‡	1.0 (0 to 23.2)	0.4 (0 to 36.3)	n.s.
DLQI‡	1.0 (0 to 20.0)	1.0 (0 to 18.0)	n.s.
DMARDs, n (%)			n.s.
MTX	33 (39.8)	30 (42.9)	
LFN	11 (13.3)	12 (17.1)	
MTX + LFN	3 (3.6)	3 (4.3)	
SSZ	2 (2.4)	2 (2.9)	
Anti-TNFα	31 (37.3)	30 (42.9)	
Corticosteroids, n (%)	6 (7.2)	4 (5.7)	n.s.
NSAIDs, n (%)			n.s.
On demand	56 (67.5)	57 (81.4)	
Regular intake	10 (12.0)	8 (11.4)	

^‡^Median (range); ^†^mean (standard deviation); *P*-values are not adjusted for multiple testing; ^¥^diagnosis was established at the day of baseline study visit. ASQol, ankylosing spondylitis quality of life; BASDAI, Bath ankylosing spondylitis disease activity index; CPDAI, composite psoriatic disease activity index; CRP, C-reactive protein (normal values 0 to 5 mg/L); DAPSA, disease activity index for psoriatic arthritis; DLQI, dermatology life quality index; DMARDs, disease-modifying anti-rheumatic drugs; EGA, evaluator’s global assessment of disease activity; ESR, erythrocyte sedimentation rate (normal values 1 to 10 mm/1^st^ hour); HAQ, health assessment questionnaire; LFN, leflunomide; MTX, methotrexate; n, number; n.a., not applicable; n.s., not statistically significant; NSAIDs, non-steroidal anti-inflammatory drugs; PASDAS, psoriatic arthritis disease activity score; PASI, psoriasis area and severity index; PGA, patients’ global assessment of disease activity; Ptpain, patients’ pain assessment; SJ, swollen joint count; SSZ, sulfasalazine; TJ, tender joint count; TNF, tumour necrosis factor alpha. PGA, Ptpain and EGA were measured on visual analogue scales (range 0 to 100 mm) and are expressed in mm; BASDAI and ASQol values are shown for those 11 (13.3%) PsA patients with axial involvement.

### Synovial recesses and entheses selected for the ultrasound composite scores

Additional file 2 details the results of selection process for the ultrasound composite scores. We made a few manual selections to improve the feasibility of the scores: (1) we included the second H-PIP instead of the first H-PIP for the PsASon22 because H-PIP2 better fitted into the construct of MCP2, MCP3, H-PIP3, H-DIP2 and H-DIP3 (that was already gathered). Also, H-PIP1 emerged from the selection process because of a high prevalence of osteophytes; however, the sensitivity of H-PIP2 for the detection of osteophytes was only slightly lower than that of H-PIP1; (2) we omitted the insertion of the Achilles tendon from both the PsASon22 and the PsASon13 scores because this site was mainly relevant for the detection of enthesophytes; and (3) we omitted the distal insertion of the triceps into the olecranon from PsASon13 because this site was mainly relevant for the detection of enthesophytes. The combination of the insertion of the common extensor tendon at the lateral epicondyle and the patellar tendon insertion into the tibial anterior tuberosity already revealed a very high sensitivity to identify patients with enthesophytes.

Table 2 summarizes the proportion of ultrasound abnormalities exclusively detected by dorsal or palmar/plantar scans of H-PIPs, H-DIPs, MTPs, F-PIPs, F-DIPs and wrists as well as by medial/lateral or suprapatellar assessments of knees. We observed that ≥20% of structural and inflammatory pathologies at the H-PIP and H-DIP level were identified by palmar or dorsal scans only. At the knees, suprapatellar, medial/lateral scans are required not to miss patients with PD-signals. At the wrists and MTPs, palmar/plantar scans appeared to be less relevant given that only a minority of patients revealed ultrasound abnormalities at these sites. At F-PIPs and F-DIPs, the proportion of erosions detected by plantar scans was >20%; however, we decided to omit plantar scans from the composite scores due to the low absolute number of erosions at these sites.

**Table 2:** **Ultrasound pathologies detected exclusively by dorsal or palmar/plantar scans of hand and foot joints and scans of different knee recesses**

	**Erosion**					**Osteophyte**					**GSS**					**PD-j**				
	**Dorsal only**		**Palmar/plantar only**		**Total**	**Dorsal only**		**Palmar/plantar only**		**Total**	**Dorsal only**		**Palmar/plantar only**		**Total**	**Dorsal only**		**Palmar/plantar only**		**Total**
**Grade (range 0 to 3)**	**1**	**2 to 3**	**1**	**2 to 3**	**1 to 3**	**1 to 3**	**2 to 3**	**1**	**1 to 3**	**1 to 3**	**1**	**1 to 3**	**1**	**2 to 3**	**1 to 3**	**1**	**2 to 3**	**1**	**2 to 3**	**1 to 3**
	**n (%)**	**n (%)**	**n (%)**	**n (%)**	**n (%)**	**n (%)**	**n (%)**	**n (%)**	**n (%)**	**n (%)**	**n (%)**	**n (%)**	**n (%)**	**n (%)**	**n (%)**	**n (%)**	**n (%)**	**n (%)**	**n (%)**	**n (%)**
**H-PIP + H-DIP**	6 (13.0)	8(17.4)	18(39.1)	12(26.1)	46(100)	117(19.5)	20(3.3)	124(20.6)	10(1.7)	601(100)	35(17.3)	14(6.9)	101(50.0)	25(12.4)	202(100)	10(22.2)	8(17.8)	14(31.1)	4(8.9)	45(100)
**MTP**	6(8.3)	32(44.4)	0	14(19.4)	72(100)	56(26.4)	49(23.1)	4(1.9)	1(0.5)	212(100)	37(42.1)	39(44.3)	3(3.5)	1(1.1)	88(100)	14(50.0)	13(46.4)	0	0	28(100)
**F-PIP + F-DIP**	8(34.8)	7(30.4)	0	6(26.1)	23(100)	74(27.8)	36(13.5)	25(9.4)	11(4.1)	266(100)	36(53.7)	9(13.4)	9(13.4)	2(3.0)	67(100)	9(37.5)	10(41.7)	1(4.2)	3(12.5)	24(100)
**Wrist**	-	-	-	-	-	-	-	-	-	-	47(51.7)	15(16.5)	1(1.1)	4(4.4)	91(100)	21(51.2)	11(26.8)	2(4.9)	0	41(100)
											**Medial/lat.**		**Suprapat.**			**Medial/lat.**		**Suprapat.**		
**Knees**											20(26.3)	9(11.8)	15(19.7)	0	76(100)	10(34.5)	9(31.0)	10(34.5)	2(6.9)	29(100)

The table depicts the number of joints at which the indicated ultrasound lesion [that is, erosions, osteophytes, grey scale synovitis (GSS) or Power Doppler signals (PD-j)] and the respective grading (1 or 2 to 3) were exclusively identified by dorsal (Dorsal only) or palmar/plantar (Palmar/plantar only) scans. Total refers to the overall number of joints positive for the specific ultrasound abnormality (irrespective of whether it was identified by dorsal, palmar/plantar or both scans). We conducted separate analyses for small finger joints except metacarpophalangeal joints (H-PIP + H-DIP), small joints of feet (MTP or F-PIP + F-DIP) and wrists. At the knees, we compared medial plus lateral (Medial/lat.) versus suprapatellar (Suprapat.) scans. Data in parenthesis reflect the percentage of positive joints out of the total number of joints revealing the indicated ultrasound finding. F-DIP, distal interphalangeal joint of the feet; F-PIP, proximal interphalangeal joint of the feet, GSS, grey scale synovitis; H-DIP, distal interphalangeal joint of hands; H-PIP, proximal interphalangeal joint of hands; MTP, metatarsophalangeal joint; PD-j, power Doppler at joints; T, total number of patients with the specific lesion; hyphens indicate “not assessed”.

**Figure 1:**
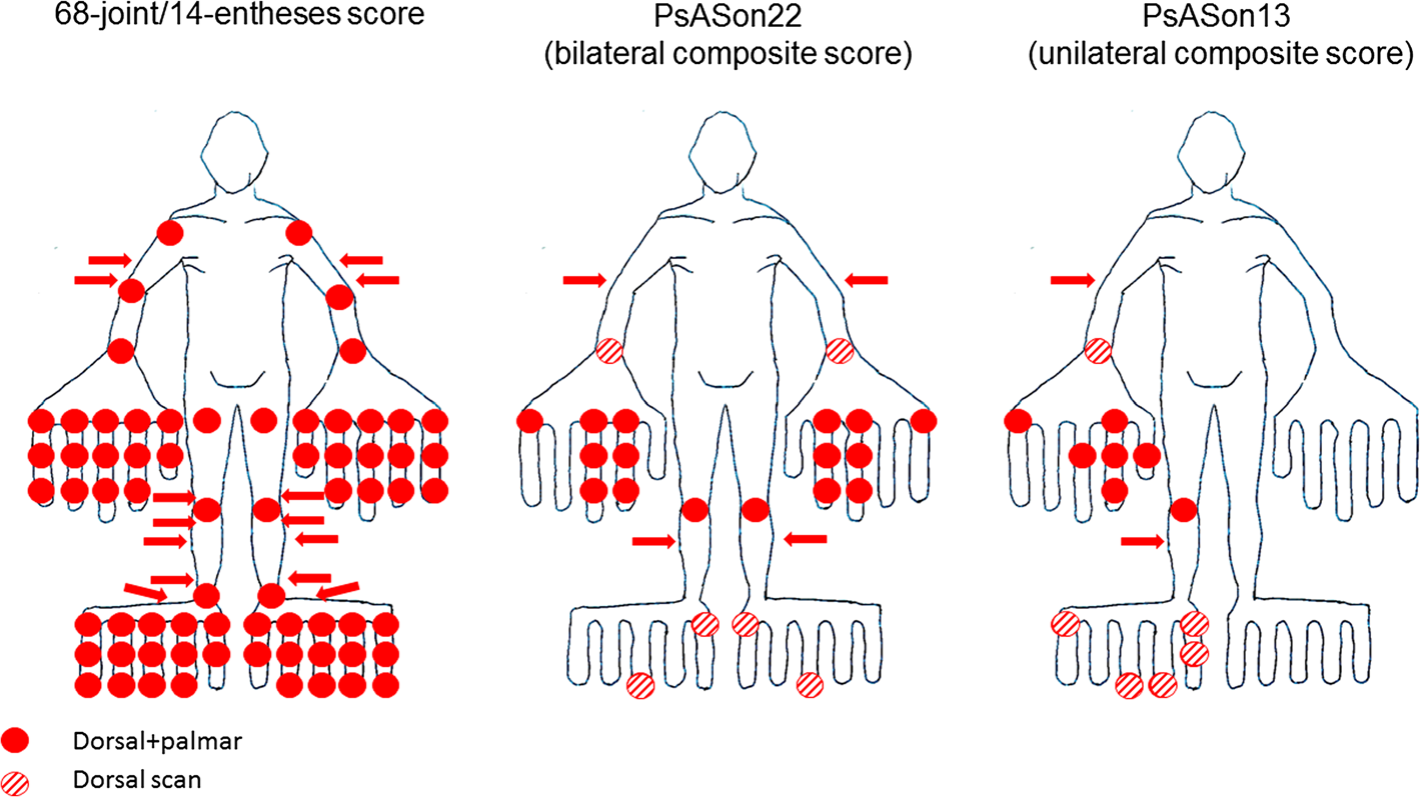
**Joints and entheses included in ultrasound composite scores.** Illustration depicts joints (circles) and entheses (arrows) included in the 68-joint/14-entheses score, and the bilateral 22-joint/4-entheses (PsASon22) and the unilateral 13-joint/2-entheses composite scores (PsASon13). For the unilateral score the dominant site is investigated (for example, the right site as shown in the figure). Solid circles indicate that both, palmar and dorsal sites (suprapatellar, medial and lateral scans for the knee) are assessed, whereas striped circles mark sites investigated by dorsal scans only.

The final composite scores are depicted in Figure 1 and detailed in Table 3. The bilateral score (PsASon22) includes 22 joints (6 MCPs, 4 H-PIPs, 2 MTPs, 4 H-DIPs, 2 F-DIPs, 4 large joints) and 4 entheses, whereas the unilateral score (PsASon13) compromises 13 joints (2 MCPs, 3 H-PIPs, 1 F-PIP, 2 MTPs, 1 H-DIP and 2 F-DIPs, 2 large joints) and 2 entheses.

**Table 3:** **Scans and anatomical sites of the bilateral (PsASon22) and unilateral ultrasound score (PsASon13)**

**PsASon22**		
**Anatomical sites**	**Scans (all bilateral)**	**Ultrasound findings**
**Small finger joints:**		
MCP2, MCP3, MCP5, H-PIP2,H-PIP3	Dorsal, palmar	GSS, PD-j, GS-Peri (MCP only), PD-Peri (MCP only), GS-Teno, PD-Teno, erosion, osteophyte
**Distal interphalangeal finger joints:**		
H-DIP2, H-DIP3	Dorsal, palmar	GSS, PD-j, GS-Teno, PD-Teno, erosion, osteophyte
**Small joints of feet:**		
MTP1	Dorsal	GSS, PD-j, erosion, osteophyte
**Distal interphalangeal joints of feet:**		
F-DIP3	Dorsal	GSS, PD-j, erosion, osteophyte
**Large joints:**		
Wrist	Dorsal	GSS, PD-j, GS-Teno (wrist only), PD-Teno (writs only)
Knee	Suppat., medial, lateral	
**Entheses:**		
Lateral epicondyle, distal patella	n.a.	GSE, PD-e, erosion, osteophyte
**PsASon13**		
**Anatomical sites**	**Scans (all unilateral, dominant site)**	**Ultrasound findings**
**Small finger joints:**		
MCP2, MCP5, H-PIP1, H-PIP2,H-PIP3	Dorsal, palmar	GSS, PD-j, GS-Peri (MCP only), PD-Peri (MCP only), GS-Teno, PD-Teno, erosion, osteophyte
**Distal interphalangeal finger joints:**		
H-DIP3	Dorsal, palmar	GSS, PD-j, GS-Teno, PD-Teno, erosion, osteophyte
**Small joints of feet:**		GSS, PD-j, erosion, osteophyte
MTP1, MTP5, F-PIP1	Dorsal	
**Distal interphalangeal joints of feet:**		
F-DIP2, F-DIP3	Dorsal	GSS, PD-j, erosion, osteophyte
**Large joints:**		
Wrist	Dorsal	GSS, PD-j, GS-Teno (wrist only), PD-Teno (writs only)
Knee	Suppat., medial, lateral	
**Entheses:**		
Lateral epicondyle, distal patella	n.a.	GSE, PD-e, erosion, osteophyte

F-DIP, Distal interphalangeal joint of feet; F-PIP, proximal interphalangeal joints of feet; GS-Peri, grey scale perisynovitis; GS-Teno, grey scale tenosynovitis; GSE, grey scale changes at entheses; GSS, grey scale synovitis; H-DIP, distal interphalangeal joint of hands; H-PIP, proximal interphalangeal joint of hands; MCP, metacarpophalangeal joint; MTP, metatarsophalangeal joint; n.a.; not applicable; PD-e, power Doppler findings at entheses; PD-j, power Doppler findings at joints; PD-Peri, power Doppler Perisynovitis PD-Teno, power Doppler Tenosynovitis; suppat., suprapatellar.

Additional file 3 details the possible ranges of ultrasound composite scores and their components.

### Sensitivity of the ultrasound composite scores for the detection of ultrasound pathologies

As detailed in Table 4, the bilateral composite score yielded sensitivity >80% for most lesions and sites using the 68-joints/14-entheses ultrasound score as the reference, whereas the unilateral score was less efficient revealing >80% sensitivity for GSS at small/large joints, GSE and osteophytes/enthesophytes only.

**Table 4:** **Sensitivity of the bilateral (PsASon22) (a) and unilateral (PsASon13) (b) ultrasound composite scores to detect PsA characteristic ultrasound findings**

**(a)**	**GSS/GSE**	**PD-j/e**	**GS-Teno**	**PD-Teno**	**GS-Peri**	**PD-Peri**	**Erosion**	**Osteop./enthesop.**
**Small joints**	89.2	81.6	71.4	75.0	95.2	92.9	91.9	100
**DIP joints**	84.6	63.6			n.a.	n.a.	42.9	98.8
**Large joints**	93.3	97.7			n.a.	n.a.	n.a.	n.a.
**entheses**	100	87.8	n.a.	n.a.	n.a.	n.a.	71.9	94.0
**(b)**	**GSS/GSE**	**PD-j/e**	**GS-Teno**	**PD-Teno**	**GS-Peri**	**PD-Peri**	**Erosion**	**Osteop./enthesop.**
**Small joints**	92.8	73.5	66.7	57.1	57.1	50.0	79.7	100
**DIPs**	58.5	54.5			n.a.	n.a.	35.7	96.4
**Large joints**	81.3	72.1			n.a.	n.a.	n.a.	n.a.
**entheses**	100	67.3	n.a.	n.a.	n.a.	n.a.	50.0	88.0

Data indicate the sensitivity of the inflammatory and structural components of the bilateral (a) and unilateral (b) ultrasound composite scores to detect ultrasound pathologies using the 68-joints/14-entheses score as a reference. DIP, distal interphalangeal joints (of hands and feet); GS-Peri, grey scale Perisynovitis; GS-Teno, grey scale tenosynovitis; GSS/GSE, grey scale synovitis/grey scale changes of entheses; n.a., pathology not assessed at this site(s); osteop./entheop., osteophytes/enthesophytes; PD-j/e, power Doppler signals at joints/entheses; PD-Peri, power Doppler Perisynovitis; PD-Teno, power Doppler Tenosynovitis; Small joints compromise metacarpophalangeal joints, metatarsophalangeal joint and proximal interphalangeal joints of hands and feet.

Both composite scores were more sensitive for the detection of PsA characteristic pathologies at small and large joints than at DIPs. Both composite scores were more efficient to identify osteophytes/enthesophytes than erosions, and among inflammatory lesions, GS-changes were more commonly detected than PD-signals.

### Convergent construct validity of ultrasound composite scores

Tables 5 and 6 depict the association of inflammatory and structural components of the bilateral and unilateral composite scores, and the 68-joint/14-entheses score with clinical parameters of inflammation and disability (convergent construct validity). We observed weak to moderate correlations of the GSS/GSE, PD-j/e, and the GUIS scores with clinical composite scores, global assessments of pain/disease activity and acute phase reactants (Table 5). Importantly, the ultrasound composite scores yielded similar associations with clinical measures of disease activity to the 68-joint/14-entheses score (except for the component PD-teno that weakly correlated with clinical composite scores only when the sonographic 68-joint/14-entheses score was applied). Joint/enthesal erosions but not osteophytes correlated with HAQ, particularly in patients judged to be in remission in whom the activity related (that is, reversible) component of disability is assumed to be low (Table 6) [20].

**Table 5:** **Correlation of the inflammatory components of the bilateral (PsASon22) and unilateral (PsASon13) ultrasound composite scores, and the 68-joint/14-entheses score with clinical parameters**

	**Score**	**PASDAS**	**CPDAI**	**DAPSA**	**Ptpain**	**PGA**	**EGA**	**CRP**	**ESR**
**GSS/GSE**	PsASon22	0.25*	NA	0.31**	NA	NA	0.29**	0.21†	0.32**
	PsASon13	0.22*	0.19†	0.28*	0.19†	NA	0.19†	0.21†	NA
	68joint/14enthes	0.33**	0.22†	0.41***	0.22*	0.24*	0.30**	0.28*	0.41***
**PD-j/e**	PsASon22	0.22*	NA	0.23*	NA	0.24*	0.22*	NA	0.37**
	PsASon13	NA	NA	NA	NA	NA	0.19†	NA	0.26*
	68joint/14enthes	0.28*	NA	0.26*	NA	0.24*	0.28*	NA	0.34**
**GS-Teno**	PsASon22	NA	NA	NA	NA	NA	NA	NA	NA
	PsASon13	NA	NA	NA	NA	NA	NA	NA	NA
	68joint/14enthes	NA	NA	NA	NA	NA	NA	NA	0.26*
**PDNATeno**	PsASon22	NA	NA	NA	NA	NA	NA	NA	0.40***
	PsASon13	NA	NA	NA	NA	NA	NA	NA	0.29**
	68joint/14enthes	0.31**	NA	0.28*	0.22*	0.28*	0.28*	0.30**	0.53***
**GS-Perisyn**	PsASon22	NA	NA	NA	NA	NA	NA	NA	NA
	PsASon13	NA	NA	NA	NA	NA	NA	NA	NA
	68joint/14enthes	NA	NA	NA	NA	NA	NA	NA	NA
**PD-Perisyn**	PsASon22	NA	NA	NA	NA	NA	NA	NA	NA
	PsASon13	NA	NA	NA	NA	NA	NA	NA	NA
	68joint/14enthes	NA	NA	NA	NA	NA	NA	NA	NA
**GUIS**	PsASon22	0.24*	NA	0.28*	NA	0.19†	0.25*	0.19†	0.35**
	PsASon13	NA	NA	0.22*	NA	NA	NA	NA	0.19†
	68joint/14enthes	0.36**	NA	0.39***	0.19†	0.28*	0.33**	0.26*	0.44***

Data indicate the correlation of the inflammatory components of the bilateral and unilateral ultrasound composite scores and the 68-joint/14-entheses score with clinical measures of disease activity. CPDAI, composite psoriatic disease activity index; CRP, C-reactive protein; DAPSA, disease activity index for psoriatic arthritis; EGA, evaluator’s global assessment of disease activity; ESR, erythrocyte sedimentation rate; GS-Peri, grey scale perisynovitis; GS-Teno, grey scale tenosynovitis; GSS/GSE, grey scale synovitis/grey scale enthesitis ultrasound score; GUIS, global ultrasound inflammation subscore; PASDAS, modified psoriatic arthritis disease activity score; PD-j/e, power Doppler scores at joints/entheses; PD-Peri, power Doppler Perisynovitis; PD-Teno, power Doppler Tenosynovitis; PGA, patients’ global assessment of disease activity; Ptpain, patients’ pain assessment. ***P <0.001; **P <0.01; *P <0.05; †*P* <0.1; NA, no significant association found.

**Table 6:** **Correlation of the structural components of the bilateral (PsASon22) and unilateral (PsASon13) ultrasound composite scores, and the 68-joint/14-entheses score with the health assessment questionnaire**

	**Score**	**HAQ**	**HAQ (clinical remission)**
**Erosion score (joint + entheses)**	PsASon22	NA	0.35*
	PsASon13	0.22*	0.39*
	68joint/14enthes	0.20†	0.34*
**Osteophyte/enthesophyte score**	PsASon22	0.20†	NA
	PsASon13	NA	NA
	68joint/14enthes	NA	NA

Data indicate the correlation of erosion and osteophyte/enthesophyte scores from bilateral, unilateral and 68-joint scores with the health assessment questionnaire (HAQ) in the total cohort (n =83) and in patients in clinical remission according to the judgment of the evaluating rheumatologist (n =40). *P <0.05; †*P* <0.1; NA, no association found.

Further subanalyses indicated that GSS and PD-j scores were associated with the number of SJ, the PD-j score with the number of TJ, and the GSE with the MASEI + epi (see Additional file 4).

### Sensitivity to change in ultrasound composite scores

**Figure 2:**
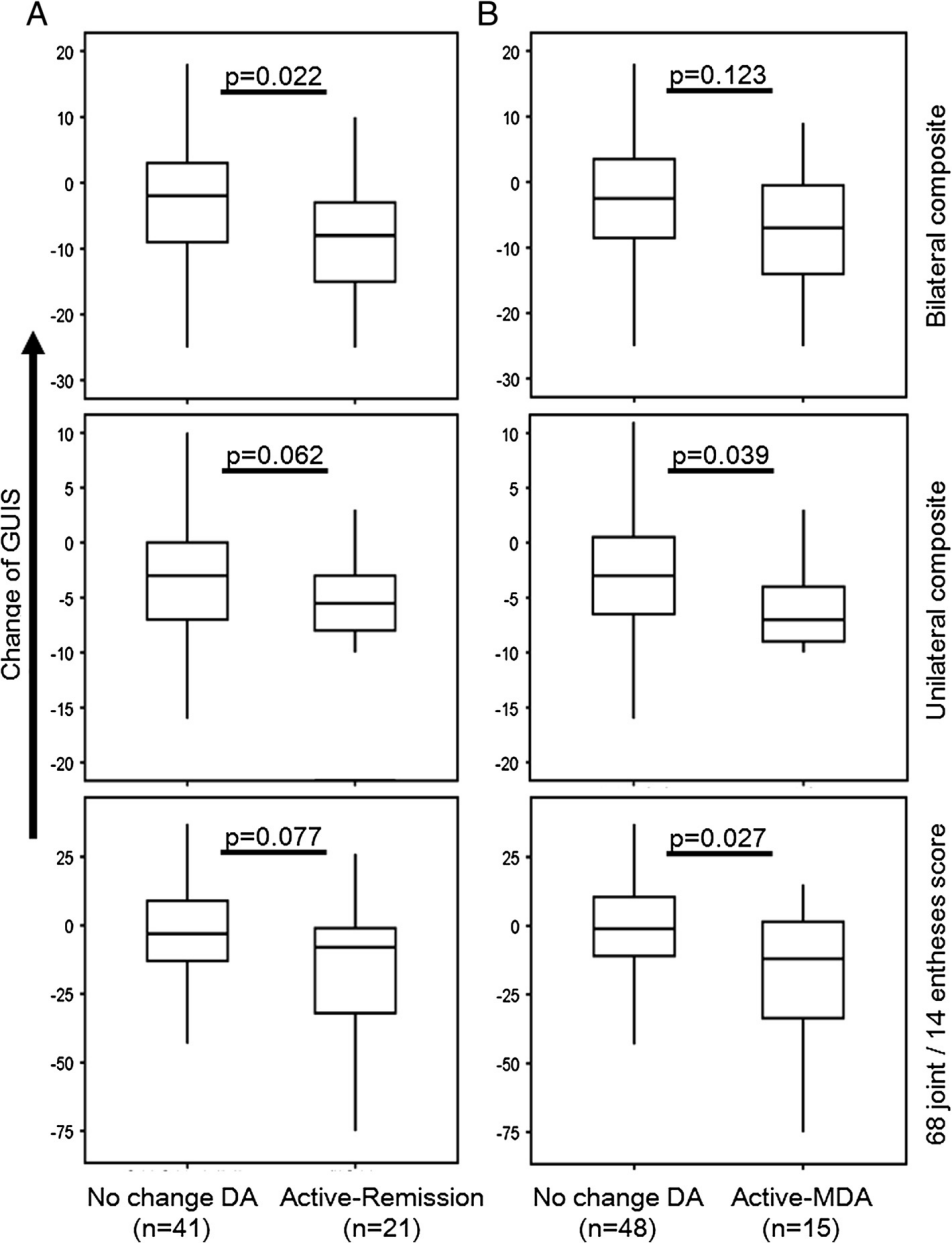
**Sensitivity to change of ultrasound composite scores.** Change (Δscore 6-month visit – score baseline) of global ultrasound inflammatory subscores (global ultrasound inflammation score (GUI-score)) in patients without a change in clinical disease activity (that is, active or remission at both baseline and follow-up visits) (no change DA) and patients who were active at baseline and achieved remission according to the evaluating physician **(A)**, active-remission) or minimal disease activity **(B)** (active-MDA) at 6 months follow-up. Whiskers box plots show the median and 50% of cases within the boxes and all data excluding mavericks between the end points of the whiskers. Differences were tested by the Mann-Whitney *U*-test.

As detailed in Figure 2 and Additional file 5 we observed that patients changing from a clinical status of active disease to remission/MDA yielded greater reductions in the GUIS of the bilateral, the unilateral or the 68-joint/14-entheses scores compared to patients with unchanged clinical activity. The effect size (Cohen’s *d* statistic) for the GUIS was low to moderate ranging from 0.40 (PsASon13) to 0.75 (68-joint/14-entheses score). SRM of patients changing from active disease to remission or MDA ranged from −1.04 to −0.09 (as compared to −0.53 to −0.04 in the entire cohort).

In subanalyses, we observed that the GSS/GSE was the most sensitive GUIS item to change (for details including effect size and SRM see Additional file 5). We also found weak to moderate correlations between ΔGUIS scores and ΔPASDAS, ΔDAPSA, ΔPtpain, ΔPGA and ΔEGA as depicted in Additional file 6.

### Feasibility and inter-rater reliability

The median time to complete the bilateral and unilateral scores was 19 minutes (range 16 to 26) and 10 (9 to 13) minutes, respectively. Inter-rater reliability of components from bilateral and unilateral composite scores was moderate to good: ICCs for the GUIS were 0.84 and 0.54, respectively. ICCs of the GUIS components ranged from 0.42 (GS-teno) to 0.96 (PD-j) for the bilateral composite score and from 0.36 (GS-teno) to 0.71 (PD-j) for the unilateral score. The ICC for erosions was 0.75 and 0.64, respectively; and for the osteophyte/enthesophyte subscore, it was 0.92 and 0.41, respectively, for the bilateral and unilateral composite scores.

## Discussion

In the present study, we propose two new ultrasound composite scores for the assessment of PsA specific inflammatory and structural lesions. Both composite scores had adequate convergent construct validity, yielded reliable results and the inflammatory elements revealed sufficient sensitivity to change. We found the bilateral score (PsASon22) more sensitive than the unilateral composite score (PsASon13) to detect PsA-specific pathologies whereas the latter was faster to accomplish.

The major strengths of our composite scores are the inclusion of all currently defined Ps-related ultrasound pathologies into the scores, and the data driven identification of joints, peri-articular structures and entheses, except for a few manual selections aimed at the improvement of the feasibility of the scores. The German U7 and the Italian five-targets scores, the two other ultrasound composite scores previously applied in PsA, were developed on a basis of clinical experience and focus on fewer sites and less PsA-specific pathologies than our instruments [5,6].

Our data demonstrate that palmar scans of wrists and plantar scans of feet are dispensable for the ultrasound composite scores, whereas the investigation of palmar and dorsal sites of fingers is required to achieve sufficient sensitivity to identify PsA-specific pathologies. Similar data were reported for the assessment of the rheumatoid hand, whereas a comparison between plantar and dorsal scans of feet has not been performed in other cohorts so far [22,23].

We did not include dactylitis into the composite scores, because of the lack of an established ultrasound definition. Earlier studies suggested that the combination of arthritis and tenosynovitis is the underlying pathology of dactylitis, whereas recent ultrasound and magnetic resonance imaging data indicate that isolated tenosynovitis, soft tissue oedema and/or collateral tendon enthesitis are frequently observed in dactylitic fingers and toes [24-28]. An outcome measures in rheumatology (OMERACT) project aimed at the agreement on a new ultrasound definition of dactylitis is currently underway, and a revision of our composite scores may be considered once such a definition has been validated [27].

MTP1 was included into the composite scores because we frequently found GSS and PD-j changes at this site. Earlier studies in PsA and RA also reported a high prevalence of inflammatory changes at MTPs; however, we recognize potential overestimation of PsA-related synovitis at that site as MTP1 is also frequently affected by osteoarthritis and because there were some cases of concomitant gout mimicking PsA flares [29-34]. We did not systematically record ultrasound signs of crystal arthropathies in our patients, but emphasise that such a study should be performed in the future [35]. Similarly, PIP and DIP joints may be inflamed either due to PsA or (secondary or concomitant) symptomatic osteoarthritis, and it is currently impossible to reliably identify the true driver of inflammation by means of imaging methods alone [36,37]. This uncertainty also relates to RA-specific ultrasound scores, and we do currently not know whether ultrasound-verified synovitis caused by the primary disease or (secondary/concomitant) osteoarthritis has a different impact on clinical and structural outcomes in RA and PsA [6,38].

We observed a higher sensitivity of the bilateral versus the unilateral composite score for the detection of inflammatory and structural changes, but at the cost of time. In situations, where a high sensitivity is less relevant (for example, use of sonography to monitor treatment response) the unilateral score may be preferred, whereas for remission assessment the bilateral ultrasound score may produce more valuable results. In RA, subclinical arthritis was of high prognostic value regarding clinical relapses and progression of erosions, and we are currently investigating the relevance of ultrasound-verified inflammation for patients’ related outcomes in PsA [39-41]. A further refinement of the ultrasound scores based on the results of this study (for example, by inclusion of sites with a high significance for the prediction of structural progression) cannot be excluded at this stage.

To test convergent construct validity, we correlated the inflammatory and structural components of the ultrasound scores with clinical measures of disease activity and disability, respectively. We applied the PASDAS, CPDAI and DAPSA as markers of clinical activity recognizing that none of these instruments have yet been established as the gold standard. HAQ better correlated with erosions than with osteophytes, whereas in RA the loss of cartilage was the most important factor contributing to patients’ disability [20].

Among individual components of the GUIS, the GSS/GSE correlated best with clinical activity and revealed the highest sensitivity to change during follow up. This observation may be related to the facts that peripheral arthritis was present in the majority of patients (74.7% had active joint disease according to CPDAI at baseline), that joint disease highly impacts clinical composite scores and global measures of disease activity and that GSS/GEE had the largest variability among the GUIS components [4,42]. Also, we observed that both ultrasound scores were generally more sensitive to detect GS than PD changes at joints and entheses, possibly explained by a high prevalence of GS-abnormalities even in PsA patients with low or no clinical activity [4].

The major limitation of our study is the relatively low clinical disease activity of the cohort leading to a possible underestimation of the sensitivity to change of the inflammatory elements. Previously proposed ultrasound scores in PsA and RA were validated in patients with high disease activity at baseline and subsequent treatment with biological agents [5,19,43]. Consequently, dramatic changes in disease scores were observed resulting in better sensitivity to change in the ultrasound composite scores by statistical means. The low disease activity in our cohort may also explain the relatively weak association between inflammatory items of the ultrasound composite scores and clinical factors. We previously concluded from an RA study that the strength of association between clinical and ultrasound measures diminishes as patients are in or close to remission [15]. Nevertheless, our ultrasound scores resulted in sufficient sensitivity to change and convergent construct validity, given that the comprehensive assessment of 68-joint/14-entheses did not produce better results than the reduced scores.

Other limitations of this study are the moderate size of the cohort, the relatively long disease duration, the single-centre design and the use of the as yet unvalidated 68-joint/14-entheses score as a reference for the development of the composite scores. Also, we did not test the responsiveness of structural components of the scores because of the short follow-up period and the absence of paired results from other imaging methods. Future studies with a long-term (preferentially multicentre) design, the inclusion of various patients’ subgroups (early disease, different clinical manifestations and new treatment with biologics) and the possibility to compare ultrasound data with other imaging methods are needed for external validation of our data.

Another interesting finding of this study is the better reliability of bilateral versus unilateral composite scores. This observation can be explained by the fact that scores with high variability of results (resulting from a larger number of sites included in the bilateral score) yield better statistical associations than scores with a narrow range [44]. The overall reproducibility of our composite scores, however, was comparable to that of previous RA- and PsA-specific scores [5,7,38].

## Conclusion

We developed two new ultrasound composite scores for PsA. Both scores include most PsA-specific pathologies, are sensitive for the detection of inflammation, have adequate convergent construct validity, are reliable and feasible in clinical practice and inflammatory elements of the scores reveal sufficient sensitivity to change.

## Additional files

Additional file 1
**Flow chart illustrating exemplarily the identification of a combination of joints for the bilateral psoriatic arthritis score (PsASon22) that is sensitive for the detection of power Doppler (PD) signals among small joints.** DIP, distal interphalangeal joints; GS-Peri, grey scale perisynovitis; GS-Teno, grey scale tenosynovitis; GSE, grey scale changes of entheses; GSS, grey scale synovitis; H-PIP, proximal interphalangeal joint of hands; MCP, metacarpophalangeal joint; MTP, metatarsophalangeal joint; osteo, osteophytes; PD-e, Power Doppler at entheses; pts, patients; step n, the procedure is repeated n-times. Additional file 2
**Results of the selection process of anatomical sites for (** **a** **) bilateral psoriatic arthritis (PsASon22) and (** **b** **) unilateral (PsASon13) ultrasound scores.**
Additional file 3
**Possible ranges in the (a) bilateral and (b) unilateral ultrasound composite scores, as well as (c) the 68-joint/14-entheses score, and their components.**
Additional file 4
**(a) Correlation of joint components from bilateral, unilateral and 68-joint scores with clinical parameters; (b) correlation of enthesal components from bilateral, unilateral and 14-entheses scores with clinical enthesis scores.**
Additional file 5
**(a) Sensitivity to change in ultrasound scores - change in clinical disease activity according to the evaluating rheumatologist; (b) sensitivity to change in ultrasound scores - change in clinical disease activity according to minimal disease activity criteria.**
Additional file 6
**Changes in ultrasound scores versus changes in clinical scores.**

## Abbreviations

CASPAR: classification for psoriatic arthritis
cMASEI + E: Madrid sonographic enthesitis index plus lateral epicondyle
CPDAI: composite psoriatic disease activity index
CRP: C-reactive protein
DAPSA: disease activitiy index for psoriatic arthritis
DIP: distal interphalangeal joint
EGA: evaluator’s global assessment of disease activity
ESR: erythrocyte sedimentation rate
EULAR: European League Against Rheumatism
F-DIP: distal interphalangeal joint of the feet
F-PIP: proximal interphalangeal joint of the feet
GS: grey scale
GSE: grey scale changes of enthesitis
GS-perisyn: grey scale perisynovitis
GSS: grey scale synovitis
GS-teno: grey scale tenosynovitis
GUIS: global ultrasound inflammation subscore
HAQ: health assessment questionnaire
H-DIP: distal interphalangeal joint of the hands
H-PIP: proximal interphalangeal joint of the hands
ICC: intraclass correlation coefficient
LEI: Leeds enthesitis index
MASEI: Madrid sonographic enthesitis index
MCP: metacarpophalangeal joint
MDA: minimal disease activity
MTP: metatarsophalangeal joint
OMERACT: outcome measures in rheumatology
PASDAS: psoriatic arthritis disease activitiy score
PASI: psoriasis activity and severity index
PD: power Doppler
PD-e: power Doppler signals at enthesis
PD-j: power Doppler signals at joints
PD-perisyn: power Doppler signals related to perisynovitis
PD-teno: power Doppler signals related to tenosynovitis
PGA: patients’ global assessment of disease activity
PIP: proximal interphalangeal joint
PsA: psoriatic Arthritis
Ptpain: patients’ pain assessment
RA: rheumatoid arthritis
SF-36: short form health survey 36
SJ: swollen joints
SRM: standardized response means
TJ: tender joints
