# Supplementary figures and images for: Ultrasound composite scores for the assessment of inflammatory and structural pathologies in Psoriatic Arthritis (PsASon-Score)
Source: Arthritis Res Ther. 2014 Oct 31;16(5):476. doi: 10.1186/s13075-014-0476-2 (PMC4247751; doi:10.1186/s13075-014-0476-2)

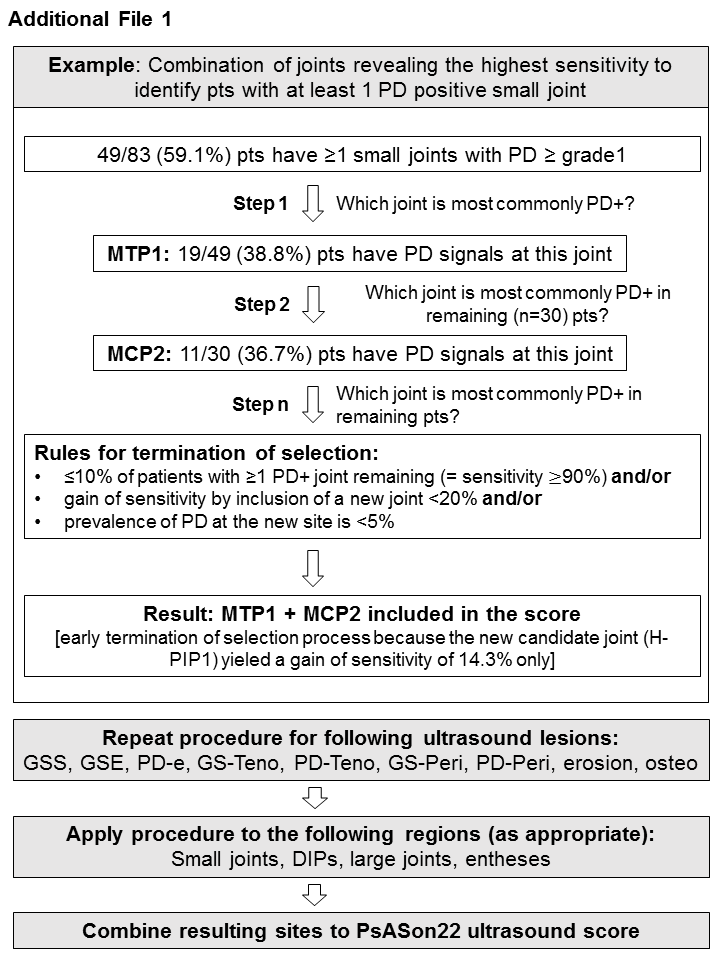

Supplement: Additional file 1 — Flow chart illustrating exemplarily the identification of a combination of joints for the bilateral psoriatic arthritis score (PsASon22) that is sensitive for the detection of power Doppler (PD) signals among small joints. DIP, distal interphalangeal joints; GS-Peri, grey scale perisynovitis; GS-Teno, grey scale tenosynovitis; GSE, grey scale changes of entheses; GSS, grey scale synovitis; H-PIP, proximal interphalangeal joint of hands; MCP, metacarpophalangeal joint; MTP, metatarsophalangeal joint; osteo, osteophytes; PD-e, Power Doppler at entheses; pts, patients; step n, the procedure is repeated n-times. [file 13075_2014_476_MOESM1_ESM.tiff]
