# Supplementary material for: Ultrasound composite scores for the assessment of inflammatory and structural pathologies in Psoriatic Arthritis (PsASon-Score)
Source: Arthritis Res Ther. 2014 Oct 31;16(5):476. doi: 10.1186/s13075-014-0476-2 (PMC4247751; doi:10.1186/s13075-014-0476-2)
Supplement: Additional file 2 — Results of the selection process of anatomical sites for ( a ) bilateral psoriatic arthritis (PsASon22) and ( b ) unilateral (PsASon13) ultrasound scores. [file 13075_2014_476_MOESM2_ESM.doc]

**Additional File 2a**. Results of the selection process of anatomical sites for the bilateral ultrasound score (PsASon22)

| **Region** | **Joint** |  | **GS** | **PD-j/e** | **B-Teno** | **PD-Teno** | **B-Peri** | **PD-Peri** | **Erosion** | **Osteophyte/**  **enthesophyte** |
| --- | --- | --- | --- | --- | --- | --- | --- | --- | --- | --- |
| **Small joints** | **MCP2** | Prevalence | 51 (30.7) | 19 (11.4) | 17 (10.2) | 3 (1.8) | 13 (7.8) | 9 (5.4) | 72 (43.4) | 130 (78.3) |
|  |  | Ranking | - | 2 | - | - | 1 | 1 | 1 | - |
|  |  | Sens gain | - | 22.5 | - | - | 52.4 | 57.1 | 68.9 | - |
|  | **MCP3** | Prevalence | 29 (17.5) | 10 (6.0) | 11 (6.6) | 3 (1.8) | 13 (7.8) | 7 (4.2)^c^ | 29 (17.5) | 112 (67.5) |
|  |  | Ranking | - | - | - | - | 1* | - | - | - |
|  |  | Sens gain | - | - | - | - | 52.4 | - | - | - |
|  | **MCP5** | Prevalence | 17 (10.2) | 7 (4.2) | 21 (12.7) | 5 (3.0)^c^ | 3 (1.8)^c^ | 1 (0.6) | 42 (25.3) | 108 (65.1) |
|  |  | Ranking | - | - | 2 | - | - |  | - | - |
|  |  | Sens gain | - | - | 30.3 | - | - |  | - | - |
|  | **H-PIP1** | Prevalence | 50 (30.1) | 20 (12.1) | 13 (7.8) | 2 (1.2) | n.a. | n.a. | 11 (6.6) | 146 (88.0)^a^ |
|  |  | Ranking | - | 3 | - | - |  |  | - | 1 |
|  |  | Sens gain | - | 14.3%^b^ | - | - |  |  | - | 94.0 |
|  | **H-PIP2** | Prevalence | 45 (27.1) | 14 (8.4) | 17 (10.2) | 4 (2.4) | n.a. | n.a. | 11 (6.6) | 133 (80.1)^d^ |
|  |  | Ranking | - | - | - | - |  |  | - | 3 |
|  |  | Sens gain | - | - | - | - |  |  | - | 91.8 |
|  | **H-PIP3** | Prevalence | 49 (29.5) | 10 (6.0) | 24 (14.5) | 6 (3.6) | n.a. | n.a. | 18 (10.8) | 126 (75.9) |
|  |  | Ranking | - | - | 1 | - |  |  | - | - |
|  |  | Sens gain | - | - | 33.9 | - |  |  | - | - |
|  | **MTP1** | Prevalence | 79 (47.6) | 26 (15.7) | 5 (3.0) | 0 | n.a. | n.a. | 43 (25.9) | 142 (85.5) |
|  |  | Ranking | 1 | 1 | - | - |  |  | - | - |
|  |  | Sens gain | 67.5 | 38.8 | - | - |  |  | - | - |
|  | **MTP5** | Prevalence | 18 (10.8) | 6 (3.6) | 2 (2.1) | 1 (0.6) | n.a. | n.a. | 55 (33.1) | 136 (81.9) |
|  |  | Ranking | - | - | - | - |  |  | 2 | - |
|  |  | Sens gain | - | - | - | - |  |  | 13.5^b^ | - |
|  | **F-PIP1** | Prevalence | 74 (44.6) | 15 (9.0) | 18 (10.8) | 1 (0.6) | n.a. | n.a. | 25 (15.1) | 130 (78.3) |
|  |  | Ranking | 2 | - | - | - |  |  | - | - |
|  |  | Sens gain | 19.3%^b^ | - | - | - |  |  | - | - |
|  | **F-PIP2** | Prevalence | 18 (10.8) | 1 (0.6) | 20 (12.1) | 2 (1.2) | n.a. | n.a. | 10 (6.0) | 126 (75.9) |
|  |  | Ranking | - | - | 3 | - |  |  | - | - |
|  |  | Sens gain | - | - | 8.5 ^b^ | - |  |  | - | - |
| **DIP-H/F** | **H-DIP2** | Prevalence | 47 (28.3) | 7 (4.2)^c^ | 13 (7.8) | 5 (3.0)^c^ | n.a. | n.a. | 6 (3.6) | 140 (84.3)^a^ |
|  |  | Ranking | 1 | 2 | 1 | 1 |  |  | - | 1 |
|  |  | Sens gain | 58.5 | - | 34.4 | - |  |  | - | 94.0 |
|  | **H-DIP3** | Prevalence | 47 (28.3) | 6 (3.6) | 8 (4.8)^c^ | 1 (0.6) | n.a. | n.a. | 4 (2.4) | 139 (83.7) |
|  |  | Ranking | 2 | - | - | - |  |  | - | - |
|  |  | Sens gain | 24.6 | - | - | - |  |  | - | - |
|  | **H-DIP4** | Prevalence | 24 (14.5) | 7 (4.2) | 7 (4.2) | 3 (1.8) | n.a. | n.a. | 7 (4.2)^c^ | 120 (72.3) |
|  |  | Ranking | - | - | - | - |  |  | - | - |
|  |  | Sens gain | - | - | - | - |  |  | - | - |
|  | **F-DIP2** | Prevalence | 22 (13.3) | 6 (3.6) | 6 (3.6) | 2 (1.2) | n.a. | n.a. | 5 (3.0) | 135 (81.3) |
|  |  | Ranking | 3 | - | - | - |  |  | - | - |
|  |  | Sens gain | 10.8^b^ | - | - | - |  |  | - | - |
|  | **F-DIP3** | Prevalence | 14 (8.4) | 10 (6.0) | 7 (4.2) | 1 (0.6) | n.a. | n.a. | 6 (3.6) | 129 (77.7) |
|  |  | Ranking | - | 1 | - | - |  |  | - | - |
|  |  | Sens gain | - | 36.4 | - | - |  |  | - | - |
| **Large joints** | **Wrist** | Prevalence | 91 (54.8) | 41 (24.7) | 6 (3.6) | 14 (8.4) | n.a. | n.a. | n.a. | n.a. |
|  |  | Ranking | 1 | 1 | - | 1 |  |  |  |  |
|  |  | Sens gain | 84.0 | 72.1 | - | 39.3 |  |  |  |  |
|  | **knee** | Prevalence | 76 (45.8) | 29 (17.5) | n.a. | n.a. | n.a. | n.a. | n.a. | n.a. |
|  |  | Ranking | 2 | 2 |  |  |  |  |  |  |
|  |  | Sens gain | 10.7^b^ | 25.6 |  |  |  |  |  |  |
|  | **ankle** | Prevalence | 24 (14.5) | 1 (0.6)^c^ | 7 (4.2)^c^ | 0^c^ | n.a. | n.a. | n.a. | n.a. |
|  |  | Ranking | - | - | - | - |  |  |  |  |
|  |  | Sens gain | - | - | - | - |  |  |  |  |
| **entheses** | **epicond** | Prevalence | 159 (95.8) | 53 (31.9) | n.a. | n.a. | n.a. | n.a. | 38 (22.9) | 126 (75.9) |
|  |  | Ranking | 1 | 1 |  |  |  |  | 1 | - |
|  |  | Sens gain | 97.6^a^ | 75.5 |  |  |  |  | 46.9 | - |
|  | **achilles** | Prevalence | 100 (60.2) | 10 (6.0) | n.a. | n.a. | n.a. | n.a. | 29 (17.5) | 144 (86.7)^e^ |
|  |  | Ranking | - | 2 |  |  |  |  | 3 | 1 |
|  |  | Sens gain | - | 10.2^b^ |  |  |  |  | 18.8%^b^ | 94.0 |
|  | **dist. pat** | Prevalence | 148 (89.2) | 9 (5.4) | n.a. | n.a. | n.a. | n.a. | 32 (19.3) | 85 (51.2) |
|  |  | Ranking | - | - |  |  |  |  | 2 | - |
|  |  | Sens gain | - | - |  |  |  |  | 25.0 | - |

**Additional File 2b**. Results of the selection process of anatomical sites for the unilateral ultrasound score (PsASon13)

| **Region** | **Joint** |  | **GS** | **PD-j/e** | **B-Teno** | **PD-Teno** | **B-Peri** | **PD-Peri** | **Erosion** | **Osteophyte/**  **enthesophyte** |
| --- | --- | --- | --- | --- | --- | --- | --- | --- | --- | --- |
| **Small joints** | **MCP2** | Prevalence | 31 (37.3) | 10 (12.1) | 10 (12.1) | 1 (1.2) | 10 (12.1) | 6 (7.2) | 43 (51.8) | 66 (79.5) |
|  |  | Ranking | 3 | 2 | - | - | 1 | 1 | 1 | - |
|  |  | Sens gain | 8.4^b^ | 16.3 | - | - | 47.6 | 42.9 | 58.1 | - |
|  | **MCP3** | Prevalence | 19 (22.9) | 6 (7.2) | 7 (8.4) | 1 (1.2) | 4 (4.8)^c^ | 3 (3.6)^c^ | 19 (22.9) | 56 (67.5) |
|  |  | Ranking | - | - | - | - | - | - | - | - |
|  |  | Sens gain | - | - | - | - |  | - | - | - |
|  | **MCP5** | Prevalence | 10 (12.1) | 3 (3.6) | 15 (18.1) | 1 (1.2) | 2 (2.4) | 1 (1.2) | 23 (27.7) | 54 (65.1) |
|  |  | Ranking | - | - | 1 | - | - | - | 3 | - |
|  |  | Sens gain | - | - | 25.4 | - | - | - | 5.4^b^ | - |
|  | **H-PIP1** | Prevalence | 26 (31.3) | 8 (9.6) | 9 (10.8) | 2 (2.4) | n.a. | n.a. | 7 (8.4) | 73 (88.0) |
|  |  | Ranking | - | 5 | 3 | - |  |  | - | 1 |
|  |  | Sens gain | - | 8.2^b^ | 11.9 | - |  |  | - | 88.0 |
|  | **H-PIP2** | Prevalence | 25 (30.1) | 11 (13.3) | 10 (12.1) | 3 (3.6)^c^ | n.a. | n.a. | 7 (8.4) | 65 (78.3) |
|  |  | Ranking | - | 4 | 4 | 1 |  |  | - | 2 |
|  |  | Sens gain | - | 10.2 | 6.8^b^ | 10.7 |  |  | - | 8.4^b^ |
|  | **H-PIP3** | Prevalence | 25 (30.1) | 3 (3.6) | 13 (15.7) | 2 (2.4) | n.a. | n.a. | 10 (12.1) | 60 (72.3) |
|  |  | Ranking | - | - | 2 | - |  |  | - | - |
|  |  | Sens gain | - | - | 18.6 | - |  |  | - | - |
|  | **MTP1** | Prevalence | 44 (53.0) | 12 (14.5) | 1 (1.2) | 0 | n.a. | n.a. | 22 (26.5) | 70 (84.3) |
|  |  | Ranking | 1 | 1 | - | - |  |  | - | - |
|  |  | Sens gain | 53.0 | 24.5 | - | - |  |  | - | - |
|  | **MTP5** | Prevalence | 9 (10.8) | 2 (2.4) | 1 (1.2) | 1 (1.2) | n.a. | n.a. | 29 (34.9) | 66 (79.5) |
|  |  | Ranking | - | - | - | - |  |  | 2 | - |
|  |  | Sens gain | - | - | - | - |  |  | 12.2 | - |
|  | **F-PIP1** | Prevalence | 39 (47.0) | 10 (12.1) | 8 (9.6) | 1 (1.2) | n.a. | n.a. | 13 (15.7) | 67 (80.7) |
|  |  | Ranking | 2 | 3 | - | - |  |  | - | - |
|  |  | Sens gain | 21.7 | 14.3 | - | - |  |  | - | - |
| **DIP-H/F** | **H-DIP2** | Prevalence | 26 (31.3) | 5 (6.0) | 9 (10.8) | 4 (4.8)^c^ | n.a. | n.a. | 3 (3.6) | 74 (89.2) |
|  |  | Ranking | 1 | 1^*^ | 1 | - |  |  | - | 1 |
|  |  | Sens gain | 40.0 | 22.7 | 29.0 | - |  |  | - | 89.2 |
|  | **H-DIP3** | Prevalence | 16 (19.3) | 3 (3.6) | 4 (4.8)^c^ | 1 (1.2) | n.a. | n.a. | 3 (3.6) | 69 (83.1) |
|  |  | Ranking | 3 | - | - | - |  |  | - | - |
|  |  | Sens gain | 6.2^b^ | - | - | - |  |  | - | - |
|  | **F-DIP2** | Prevalence | 14 (16.9) | 4 (4.8)^c^ | 3 (3.6) | 1 (1.2) | n.a. | n.a. | 4 (4.8)^c^ | 70 (84.3) |
|  |  | Ranking | 2 | - | - | - |  |  | - | - |
|  |  | Sens gain | 16.9 | - | - | - |  |  | - | - |
|  | **F-DIP3** | Prevalence | 5 (6.0) | 5 (6.0) | 3 (3.6) | 0 | n.a. | n.a. | 4 (4.8) | 65 (78.3) |
|  |  | Ranking | - | 1 | - | - |  |  | - | - |
|  |  | Sens gain | - | 22.7 | - | - |  |  | - | - |
|  | **F-DIP4** | Prevalence | 6 (7.2) | 1 (1.2) | 0 | 0 | n.a. | n.a. | 4 (4.8) | 70 (84.3) |
|  |  | Ranking | - | - | - | - |  |  | - | 2 |
|  |  | Sens gain | - | - | - | - |  |  | - | 7.2^b^ |
| **Large joints** | **Wrist** | Prevalence | 47 (56.6) | 20 (24.1) | 3 (3.6)^c^ | 9 (10.8) | n.a. | n.a. | n.a. | n.a. |
|  |  | Ranking | 1 | 1 | - | 1 |  |  |  |  |
|  |  | Sens gain | 62.7 | 46.5 | - | 32.1 |  |  |  |  |
|  | **knee** | Prevalence | 37 (44.6) | 17 (20.5) | n.a. | n.a. | n.a. | n.a. | n.a. | n.a. |
|  |  | Ranking | 2 | 2 |  |  |  |  |  |  |
|  |  | Sens gain | 20.0 | 25.6 |  |  |  |  |  |  |
|  | **ankle** | Prevalence | 16 (19.3) | 0^c^ | 2 (2.4) | 0^c^ | n.a. | n.a. | n.a. | n.a. |
|  |  | Ranking | 3 | - | - | - |  |  |  |  |
|  |  | Sens gain | 5.3^b^ | - | - | - |  |  |  |  |
| **entheses** | **Triceps** | Prevalence | 63 (75.9) | 4 (4.8) | n.a. | n.a. | n.a. | n.a. | 12 (14.5) | 66 (79.5)^f^ |
|  |  | Ranking | - | - |  |  |  |  | - | 2 |
|  |  | Sens gain | - | - |  |  |  |  | - | 12.0 |
|  | **epicond** | Prevalence | 79 (95.2) | 29 (34.9) | n.a. | n.a. | n.a. | n.a. | 18 (21.7) | 65 (78.3) |
|  |  | Ranking | 1 | 1 |  |  |  |  | 1 |  |
|  |  | Sens gain | 95.2^a^ | 59.2 |  |  |  |  | 28.1 | - |
|  | **achilles** | Prevalence | 69 (83.1) | 4 (4.8) | n.a. | n.a. | n.a. | n.a. | 14 (16.9) | 70 (84.3)^e^ |
|  |  | Ranking | - | - |  |  |  |  | 3 | 1 |
|  |  | Sens gain | - | - |  |  |  |  | 9.5^b^ | 84.3 |
|  | **dist. pat** | Prevalence | 75 (90.4) | 6 (7.2) | n.a. | n.a. | n.a. | n.a. | 17 (20.5) | 43 (51.8) |
|  |  | Ranking | - | 2 |  |  |  |  | 2 | - |
|  |  | Sens gain | - | 8.2^b^ |  |  |  |  | 21.9 | - |

We used a hierarchical approach to identify relevant sites for the ultrasound scores as detailed in Materials and Methods and exemplarily illustrated in Additional File 1. “Prevalence” indicates the total number of sites with the corresponding ultrasound finding (e.g. GS, PD-j/e ect.; with a possible maximum of 166 sites for the bilateral score because of bilateral assessment of 83 patients and a maximum of 83 sites for the unilateral score) and the percentage in parenthesis (calculated as n/166*100% and n/83*100%, respectively). “Ranking” reflects the order of adding sites to the score. We generated rankings for each ultrasound abnormality (i.e. GS, PD-j/e ect.) and each anatomical construct (i.e. small joints, DIP-H/F, large joints and entheses) separately. “Sens gain” indicates the gain of sensitivity achieved by the inclusion of a new site to a given combination of joints/entheses for the detection of ultrasound abnormalities (i.e. GS, PD-j/e ect.) as compared to the 68-joint/14-entheses score.

The reasons for termination of the selection process were: a combination of joints or entheses reached a ≥90% sensitivity to detect the specific ultrasound abnormality (i.e. GS, PD-j/e ect.) among the anatomical construct (i.e. small joints, DIP-H/F, large joints and entheses) as compared to the 68-joint/14-entheses score (a); the gain of sensitivity by the inclusion of a new site was <20% for the bilateral score and <10% for the unilateral score (b); the prevalence of the finding at the new site was <5% (c).

We made a few manual selections to improve the feasibility of the composite scores: we included 2^nd^  H-PIP instead of 1^st^ H-PIP for the PsASon22 because H-PIP2 better fitted into the construct of MCP2, MCP3, H-PIP3, H-DIP2 and H-DIP3. H-PIP1 was relevant for detection of osteophytes only and the sensitivity of H-PIP2 for detection of osteophytes was similar to that of H-PIP1 (d). We omitted the Achilles tendon from both, the bilateral and the unilateral composite scores because this site was mainly relevant for the detection of enthesophytes and the combination of lateral epicondyle and distal patellar tendon already revealed sensitivities of 94.0% and 88%, respectively to identify patients with enthesophytes (e). We omitted the insertion of the Triceps tendon from the unilateral score for the same reason (f).

Achilles, Achilles tendon insertion B-Peri, B-Perisynovitis; B-Teno, B-Tenosynovitis; DIP-H/F, distal interphalangeal joints of hands and feet; dist. pat, insertion of the distal patellar tendon; epicond, lateral epicondyle; F-DIP, distal interphalangeal joints of feet; F-PIP, proximal interphalangeal joints of feet; GS, grey scale synovitis (of joints) / grey scale changes of entheses; H-DIP, distal interphalangeal joint of hands; H-PIP, proximal interphalangeal joint of hands; MCP, metacarpophalangeal joints; MTP, metatarsophalangeal joints; n.a., not applicable; PD-j/e, Power Doppler signals at joints/entheses, PD-Peri, Power Doppler Perisynovitis; PD-Teno, Power Doppler Tenosynovitis; Small joints compromise metacarpophalangeal joints, metatarsophalangeal joint and proximal interphalangeal joints of hands and feet; *both sites rank first
