# Supplementary material for: Ultrasound composite scores for the assessment of inflammatory and structural pathologies in Psoriatic Arthritis (PsASon-Score)
Source: Arthritis Res Ther. 2014 Oct 31;16(5):476. doi: 10.1186/s13075-014-0476-2 (PMC4247751; doi:10.1186/s13075-014-0476-2)
Supplement: Additional file 3 — Possible ranges in the (a) bilateral and (b) unilateral ultrasound composite scores, as well as (c) the 68-joint/14-entheses score, and their components. [file 13075_2014_476_MOESM3_ESM.doc]

**Additional File 3.** Possible ranges of the (a) bilateral (PsASon22) and (b) unilateral (PsASon13) ultrasound composite scores as well as the (c) 68-joint/14-entheses score and their components

| **(a)** | **GSS/GSE** | **PD-j/e** | **GS-Teno** | **PD-Teno** | **GS-Peri** | **PD-Peri** | **Erosion** | **Osteophyte** |
| --- | --- | --- | --- | --- | --- | --- | --- | --- |
| **Small joints** | 0-36 | 0-36 | 0-24 | 0-60 | 0-6 | 0-6 | 0-36 | 0-36 |
| **DIPs** | 0-18 | 0-18 |  |  | n.a. | n.a. | 0-18 | 0-18 |
| **Large joints** | 0-12 | 0-12 |  |  | n.a. | n.a. | n.a. | n.a. |
| **entheses** | 0-10 | 0-12 | n.a. | n.a. | n.a. | n.a. | 0-12 | 0-12 |
| **Total** | 0-76 | 0-78 | 0-24 | 0-60 | 0-6 | 0-6 | 0-66 | 0-66 |
| **GUIS** | 0-250 | | | | | | n.a. | n.a. |

| **(b)** | **GSS/GSE** | **PD-j/e** | **GS-Teno** | **PD-Teno** | **GS-Peri** | **PD-Peri** | **Erosion** | **Osteophyte** |
| --- | --- | --- | --- | --- | --- | --- | --- | --- |
| **Small joints** | 0-24 | 0-24 | 0-14 | 0-36 | 0-2 | 0-2 | 0-24 | 0-24 |
| **DIPs** | 0-9 | 0-9 |  |  | n.a. | n.a. | 0-9 | 0-9 |
| **Large joints** | 0-6 | 0-6 |  |  | n.a. | n.a. | n.a. | n.a. |
| **entheses** | 0-5 | 0-6 | n.a. | n.a. | n.a. | n.a. | 0-6 | 0-6 |
| **Total** | 0-44 | 0-45 | 0-14 | 0-36 | 0-2 | 0-2 | 0-39 | 0-39 |
| **GUIS** | 0-143 | | | | | | n.a. | n.a. |

| **(c)** | **GSS/GSE** | **PD-j/e** | **GS-Teno** | **PD-Teno** | **GS-Peri** | **PD-Peri** | **Erosion** | **Osteophyte** |
| --- | --- | --- | --- | --- | --- | --- | --- | --- |
| **Small joints** | 0-120 | 0-120 | 0-68 | 0-180 | 0-8 | 0-8 | 0-120 | 0-120 |
| **DIPs** | 0-48 | 0-48 |  |  | n.a. | n.a. | 0-48 | 0-48 |
| **Large joints** | 0-36 | 0-36 |  |  | n.a. | n.a. | n.a. | n.a. |
| **Entheses** | 0-32 | 0-42 | n.a. | n.a. | n.a. | n.a. | 0-42 | 0-42 |
| **Total** | 0-236 | 0-246 | 0-68 | 0-180 | 0-8 | 0-8 | 0-210 | 0-210 |
| **GUIS** | 0-746 | | | | | | n.a. | n.a. |

DIP, distal interphalangeal joints (of hands and feet); GS-Peri, grey scale perisynovitis; GS-Teno, grey scale tenosynovitis; GSS/GSE, grey scale synovitis at joints and grey scale changes at entheses; GUIS, global ultrasound inflammation sub-score (see Materials and Methods for calculation); n.a., not applicable; PD-j/e, Power Doppler scores at joints/entheses; PD-Peri, PD-Perisynovitis; PD-Teno, PD-Tenosynovitis; Small joints, small joints include metacarpophalangeal joints, metatarsophalangeal joints and proximal interphalangeal joints of hands and feet;
