# Supplementary material for: Ultrasound composite scores for the assessment of inflammatory and structural pathologies in Psoriatic Arthritis (PsASon-Score)
Source: Arthritis Res Ther. 2014 Oct 31;16(5):476. doi: 10.1186/s13075-014-0476-2 (PMC4247751; doi:10.1186/s13075-014-0476-2)
Supplement: Additional file 4 — (a) Correlation of joint components from bilateral, unilateral and 68-joint scores with clinical parameters; (b) correlation of enthesal components from bilateral, unilateral and 14-entheses scores with clinical enthesis scores. [file 13075_2014_476_MOESM4_ESM.doc]

**Additional File 4a.** Correlation of joint components from bilateral, unilateral and 68-joint scores with clinical parameters

|  | **Score** | **SJ** | **TJ** |
| --- | --- | --- | --- |
| **GSS** | bilateral | 0.34** | - |
|  | unilateral | 0.36** | - |
|  | 68-joint | 0.44*** | 0.20† |
| **PD-j** | bilateral | 0.39*** | 0.25* |
|  | unilateral | 0.43*** | 0.28* |
|  | 68-joint | 0.48*** | 0.30** |

Data indicate the correlation of grey scale synovitis (GSS) and Power Doppler scores at joints (PD-j) from bilateral, unilateral and 68-joint scores with tender (TJ) and swollen joint (SJ) counts

***p<0.001; **p<0.01; *p<0.05; †p<0.1; -, no association found

**Additional File 4b.** Correlation of enthesal components from bilateral, unilateral and 14-entheses scores with clinical enthesis scores

|  | **Score** | **Leeds** | **Clinical MASEI+E** |
| --- | --- | --- | --- |
| **GS-entheso** | bilateral | - | 0.19† |
|  | unilateral | - | - |
|  | 14 entheses | - | 0.20† |
| **PD-entheso** | bilateral | - | - |
|  | unilateral | - | - |
|  | 14 entheses | - | - |

Data indicate the correlation coefficients of grey scale enthesis sub-score (GS-entheso) and Power Doppler enthesis subscore (PD-entheso) with the clinical composites Leeds clinical enthesitis index (Leeds) and the clinical version of the Madrid Sonographic Enthesis Index+lateral epicondyle (clinical MASEI+Ei)

†p<0.1; -, no significant association found
