# Supplementary material for: Ultrasound composite scores for the assessment of inflammatory and structural pathologies in Psoriatic Arthritis (PsASon-Score)
Source: Arthritis Res Ther. 2014 Oct 31;16(5):476. doi: 10.1186/s13075-014-0476-2 (PMC4247751; doi:10.1186/s13075-014-0476-2)
Supplement: Additional file 5 — (a) Sensitivity to change in ultrasound scores - change in clinical disease activity according to the evaluating rheumatologist; (b) sensitivity to change in ultrasound scores - change in clinical disease activity according to minimal disease activity criteria. [file 13075_2014_476_MOESM5_ESM.doc]

**Additional File 5a** Sensitivity to change of the ultrasound composite scores (based on the change of clinical disease activity according to the judgment of the evaluating rheumatologist)

|  | **Score** | **no change DA**  **(n=41)** | **active-remission (n=21)** | **p-value** | **Cohen** | **SRM** | |
| --- | --- | --- | --- | --- | --- | --- | --- |
|  |  |  |  |  |  | Total cohort | active-remission |
| **GSS/**  **GSE** | **PsASon22** | **0 (-10 to 10)** | **-3 (-19 to 3)** | **0.008** | **0.79** | **-0.37** | **-0.86** |
|  | **PsASon13** | **0 (-8 to 7)** | **-2 (-13 to 3)** | **0.026** | **0.57** | **-0.21** | **-0.66** |
|  | **68-j/14-e** | **0 (-8 to 7)** | **-2 (-13 to 3)** | **0.026** | **0.43** | **-0.08** | **-0.49** |
| **PD-j/e** | PsASon22 | -1 (-16 to 8) | -2 (-14 to 5) | 0.68 | 0.13 | -0.34 | -0.50 |
|  | PsASon13 | 0 (-8 to 7) | -2 (-13 to 3) | 0.60 | 0.10 | -0.24 | -0.46 |
|  | 68-j/14-e | 2 (-26 to 29) | -4 (-41 to 16) | 0.92 | 0.21 | -0.35 | -0.44 |
| **GS-Teno** | PsASon22 | 0 (-6 to 5) | 0 (-6 to 3) | 0.30 | 0.35 | -0.18 | -0.39 |
|  | PsASon13 | 0 (-4 to 5) | 0 (-4 to 1) | 0.15 | 0.39 | -0.24 | -0.57 |
|  | 68-j/14-e | 0 (-10 to 8) | 0 (-9 to 3) | 0.32 | 0.26 | -0.28 | -0.54 |
| **PD-Teno** | PsASon22 | 0 (-6 to 3) | 0 (-9 to 8) | 0.32 | 0.08 | -0.04 | -0.09 |
|  | PsASon13 | 0 (-4 to 6) | 0 (-6 to 1) | 0.29 | 0.28 | -0.06 | -0.34 |
|  | 68-j/14-e | 0 (-7 to 6) | 0 (-15 to 11) | 0.12 | 0.35 | -0.15 | -0.30 |
| **GS-Peri** | PsASon22 | 0 (-3 to 2) | 0 (-2 to 1) | 0.15 | 0.40 | -0.31 | -0.59 |
|  | PsASon13 | 0 (-1 to 1) | 0 (-1 to 1) | 0.21 | 0.31 | -0.22 | -0.37 |
|  | 68-j/14-e | 0 (-3 to 2) | 0 (-2 to 1) | 0.16 | 0.34 | -0.27 | -0.59 |
| **PD-Peri** | PsASon22 | 0 (-1 to 1) | 0 (-2 to 1) | 0.17 | 0.45 | -0.24 | -0.42 |
|  | PsASon13 | 0 (-1 to 0) | 0 (-1 to 1) | 0.78 | 0.06 | -0.21 | -0.13 |
|  | 68-j/14-e | 0 (-1 to 1) | 0 (-2 to 1) | 0.17 | 0.45 | -0.24 | -0.42 |
| **GUIS** | **PsASon22** | **-2 (-25 to 18)** | **-8 (-25 to 10)** | **0.022** | **0.62** | **-0.43** | **-0.89** |
|  | PsASon13 | -3 (-22 to 11) | -6 (-23 to 30) | 0.062 | 0.43 | -0.53 | -0.93 |
|  | 68-j/14-e | -3 (-43 to 37) | -8 (-75 to 26) | 0.077 | 0.58 | -0.27 | -0.60 |

Data indicate the median (range) change (Δ score at 6-month visit minus score at baseline visit) of the bilateral (PsASon22), unilateral (PsASon13) and 68-joint/14-entheses scores (68-j/14-e) in patients without a change of clinical disease activity (i.e. active at baseline and follow-up or remission at baseline and follow-up, n=41) according to the evaluating physician (no change DA) and patients who were active at baseline and achieved remission at 6-months follow-up (active-remission, n=21). Statistically significant results are depicted in bold letters/number. We also calculated Cohen’s d effect size statistic (Cohen) as well as the standardized response means (SRMs) for the entire cohort (n=62) and the “active-remission” group (n=21).

**Additional File 5b** Sensitivity to change of the ultrasound composite scores (based on the change of clinical disease activity according to minimal disease activity criteria)

|  | **Score** | **no change DA**  **(n=48)** | **Active-MDA**  **(n=15)** | **p-value** | **Cohen** | **SRM** | |
| --- | --- | --- | --- | --- | --- | --- | --- |
|  |  |  |  |  |  | total cohort | active-MDA |
| **GSS/**  **GSE** | PsASon22 | -1 (-19 to 10) | -2 (-12 to 4) | 0.111 | 0.49 | -0.37 | -0.69 |
|  | **PsASon13** | **0 (-13 to 7)** | **-3 (-6 to 4)** | **0.012** | **0.65** | **-0.21** | **-0.85** |
|  | **68-j/14-e** | **2.5 (-41 to 29)** | **-4 (-33 to 5)** | **0.013** | **0.60** | **-0.08** | **-0.69** |
| **PD-j/e** | PsASon22 | -0.5 (-16 to 12) | -3 (-13 to 4) | 0.24 | 0.28 | -0.34 | -0.66 |
|  | PsASon13 | 0 (-11 to 10) | -2 (-10 to 3) | 0.34 | 0.28 | -0.24 | -0.51 |
|  | 68-j/14-e | 0 (-20 to 12) | -4 (-34 to 7) | 0.071 | 0.52 | -0.35 | -0.63 |
| **GS-Teno** | PsASon22 | 0 (-6 to 5) | 0 (-5 to 4) | 0.97 | 0.10 | -0.18 | -0.13 |
|  | PsASon13 | 0 (-4 to 5) | 0 (-4 to 2) | 0.87 | 0.10 | -0.24 | -0.29 |
|  | 68-j/14-e | 0 (-10 to 6) | -2 (-7 to 5) | 0.69 | 0.10 | -0.28 | -0.40 |
| **PD-Teno** | PsASon22 | 0 (-6 to 8) | 0 (-9 to 1) | 0.57 | 0.27 | -0.04 | -0.29 |
|  | PsASon13 | 0 (-4 to 6) | 0 (-6 to 0) | 0.25 | 0.31 | -0.06 | -0.34 |
|  | 68-j/14-e | 0 (-7 to 11) | 0 (-15 to 1) | 0.30 | 0.58 | -0.15 | -0.49 |
| **GS-Peri** | PsASon22 | 0 (-3 to 2) | 0 (-2 to 0) | 0.89 | 0 | -0.31 | -0.36 |
|  | PsASon13 | 0 (-1 to 1) | - | n.a. | n.a. | n.a. | n.a. |
|  | 68-j/14-e | 0 (-3 to 2) | 0 (-2 to 0) | 0.96 | 0 | -0.27 | -0.36 |
| **PD-Peri** | PsASon22 | 0 (-2 to 1) | 0 (-2 to 1) | 0.88 | 0.19 | -0.24 | -0.26 |
|  | PsASon13 | 0 (-1 to 1) | - | n.a. | n.a. | n.a. | n.a. |
|  | 68-j/14-e | 0 (-2 to 1) | 0 (-2 to 1) | 0.88 | 0.19 | -0.24 | -0.26 |
| **GUIS** | PsASon22 | -2.5 (-25 to 18) | -7 (-25 to 9) | 0.123 | 0.45 | -0.43 | -0.79 |
|  | **PsASon13** | **-3 (-23 to 11)** | **-7 (-21 to 5)** | **0.039** | **0.40** | **-0.53** | **-1.04** |
|  | **68-j/14-e** | **-1 (-46 to 37)** | **-12 (-75 to 15)** | **0.027** | **0.75** | **-0.27** | **-0.74** |

Data indicate the median (range) change (Δ score at 6-month visit minus score at baseline visit) of the bilateral (PsASon22), unilateral (PsASon13) and 68-joint/14-entheses scores in patients without a change of clinical disease activity [i.e. active at baseline and follow-up or minimal disease activity (MDA) at baseline and follow-up, n=48] and patients who were active at baseline and achieved MDA at 6 months follow-up (active-MDA, n=15). Statistically significant results are depicted in bold letters/number. We also calculated Cohen’s d effect size statistic (Cohen) as well as the standardized response means (SRMs) for the entire cohort (n=63) and the “active-MDA” group (n=15).

GS-Peri, grey scale perisynovitis; GS-Teno, grey scale tenosynovitis; GSS/GSE, grey scale synovitis at joints and grey scale changes at entheses; GUIS, global ultrasound inflammation sub-score (see Materials and Methods for calculation); n.a., not applicable; PD-j/e, Power Doppler scores at joints/entheses; PD-Peri, PD-Perisynovitis; PD-Teno, PD-Tenosynovitis
