# Supplementary material for: Ultrasound composite scores for the assessment of inflammatory and structural pathologies in Psoriatic Arthritis (PsASon-Score)
Source: Arthritis Res Ther. 2014 Oct 31;16(5):476. doi: 10.1186/s13075-014-0476-2 (PMC4247751; doi:10.1186/s13075-014-0476-2)
Supplement: Additional file 6 — Changes in ultrasound scores versus changes in clinical scores. [file 13075_2014_476_MOESM6_ESM.doc]

**Additional File 6** Changes of ultrasound scores versus changes of clinical scores

|  | **ΔScore** | **ΔPASDAS** | **ΔCPDAI** | **ΔDAPSA** | **ΔPGA** | **ΔPtpain** | **ΔEGA** | **ΔCRP** | **ΔESR** |
| --- | --- | --- | --- | --- | --- | --- | --- | --- | --- |
| **GSS/**  **GSE** | PsASon22 | - | - | 0.23† |  |  | 0.34** | - | 0.28† |
|  | PsASon13 | 0.24† | - | 0.24† | 0.30* | 0.38** | 0.23† | - | 0.37* |
|  | 68-joint/14-entheses | 0.33* | - | 0.41** | 0.31** | 0.39** | 0.30* | 0.24† | 0.41** |
| **PD-j/e** | PsASon22 | - | - | 0.22† | 0.23† | - | 0.24* | - | - |
|  | PsASon13 | - | - | - | 0.24* | - | 0.20† | - | - |
|  | 68-joint/14-entheses | 0.23† | - | - | 0.28* | - | 0.22† | - | - |
| **GS-Teno** | PsASon22 | - | - | - | - | - | - | - | - |
|  | PsASon13 | - | - | - | - | - | - | - | - |
|  | 68-joint/14-entheses | - | - | - | - | - | - | - | - |
| **PD-Teno** | PsASon22 | - | - | - | - | - | - | - | - |
|  | PsASon13 | - | - | - | - | - | - | - | - |
|  | 68-joint/14-entheses | 0.35** | 0.30* | 0.40** | 0.28* | 0.33** | 0.25* | - | 0.41** |
| **GS-Peri** | PsASon22 | -0.22† | - | - | - | - | - | - | - |
|  | PsASon13 | - | -0.28* | - | - | - | - | - | - |
|  | 68-joint/14-entheses | - | - | - | - | - | - | - | - |
| **PD-Peri** | PsASon22 | - | - | - | - | - | - | - | -0.34* |
|  | PsASon13 | - | - | - | - | - | - | - | -0.35* |
|  | 68-joint/14-entheses | - | - | - | - | - | - | - | -0.34* |
| **GUIS** | PsASon22 | - | - | 0.21† | - | - | 0.30* | - | - |
|  | PsASon13 | 0.21† | - | 0.27* | 0.32** | 0.28* | 0.23† | - | - |
|  | 68-joint/14-entheses | 0.31* | - | 0.37** | 0.32** | 0.35** | 0.32** | - | 0.29† |

Data indicate the correlation between the change (Δ score at 6-months visit minus score at baseline) of the bilateral (PsASon22), unilateral (PsASon13) or 68-joint/14-entheses scores with the change (Δ score at 6-months visit minus score at baseline) of clinical disease activity measures.

CPDAI, composite psoriatic disease activity index; CRP, C-reactive protein; DAPSA, Disease Activity index for PSoriatic Arthritis; EGA, evaluator’s global assessment of disease activity; ESR, erythrocyte sedimentation rate; GS-Peri, greay scale perisynovitis; GS-Teno, greay scale tenosynovitis; GSS/GSE, grey scale synovitis at joints and grey scale changes at entheses; GUIS, global ultrasound inflammation sub-score (see Materials and Methods for calculation); PASDAS, modified Psoriatic ARthritis Disease Activity Score; PD-j/e, Power Doppler scores at joints/entheses; PD-Peri, PD-Perisynovitis; PD-Teno, PD-Tenosynovitis; PGA, patients’ global assessment of disease activity; Ptpain, patients’ pain assessment;

***p<0.001; **p<0.01; *p<0.05; †p<0.1; -, no association found
